# Risk Factors of Diarrhoea among Children Under Five Years in Southwest Nigeria

**DOI:** 10.1155/2021/8868543

**Published:** 2021-02-26

**Authors:** Harriet U. Ugboko, Obinna C. Nwinyi, Solomon U. Oranusi, Fasina F. Fagbeminiyi

**Affiliations:** ^1^Department of Biological Sciences, Covenant University, Ota, Nigeria; ^2^Department of Economics and Development Studies, Covenant University, Ota, Nigeria

## Abstract

Diarrhoea is the passage of three or more loose or liquid stools per day or more frequent passage than is normal for an individual. Diarrhoea alters the microbiome, thus the immune system, and is a significant cause of morbidity and mortality in young children. This study evaluated the association between the risk factors and diarrhoea prevalence among children under five years in Lagos and Ogun States, located in Southwest Nigeria. Participants included 280 women aged 15–49 years and children aged 0–59 months. The study used quantitative data, which were assessed by a structured questionnaire. Data obtained were analyzed using the Statistical Package for the Social Sciences Software Version 25.0 and Microsoft Excel 2013. The relationships and/or association between variables were evaluated using Pearson's Chi Square and logistic regression tests. One hundred and eighteen (42%) of the children were male, and 162 (58%) were female. The majority of the children belonged to the age group 0–11 months (166). Age (*p*=0.113) and gender (*p*=0.366) showed no significant association with diarrhoea among the children. The majority of the mothers belonged to the age group 30–34. Multivariate analysis showed that the mother's level of education (95% CI for OR = 11.45; *P*=0.0001) and family income (95% CI for OR = 7.61, *P*=0.0001) were the most significant risk factors for diarrhoea among children. Mother's educational status, mother's employment, and family income were the factors significantly associated with diarrhoea in Southwest Nigeria. The study recommends that female education should be encouraged by the right government policy to enhance the achievement of the sustainable development goal three (SDG 3) for the possible reduction of neonates and infants' deaths in Nigeria.

## 1. Introduction

World Health Organisation defines diarrhoea as “the passage of three or more loose or liquid stools per day or more frequent passage than is normal for the individual” [1, 2]. Diarrhoeal disease is the second leading cause of death in children under five years old and is responsible for killing around 525 000 children every year [2]. Childhood diarrhoea affecting children five years old and below accounts for approximately 63% of diarrhoea burden [3, 4] and is the second significant cause of infant mortality in developing nations [2, 5–7], where poor sanitation and insufficient potable water are lacking [8, 9]. In Southwest Nigeria, diarrhoea is one of the three most prevalent water-related diseases, the others being typhoid fever and cholera [10, 11]. In Southwest Nigeria, most of the studies on childhood diarrhoea have focused on the molecular epidemiology of diarrhoeagenic agents [12–15]. Several other studies have reported the antimicrobial activities of indigenous medicinal plants on diarrhoea-causing agents [16–18]. However, there are insufficient reports on the associated risk factors of childhood diarrhoea in Southwest Nigeria [19, 20]. Therefore, this study aimed to determine the risk factors associated with diarrhoea prevalence among children under age 5 in Lagos and Ogun States, Nigeria.

## 2. Methods

### 2.1. Study Area

**Figure 1:**
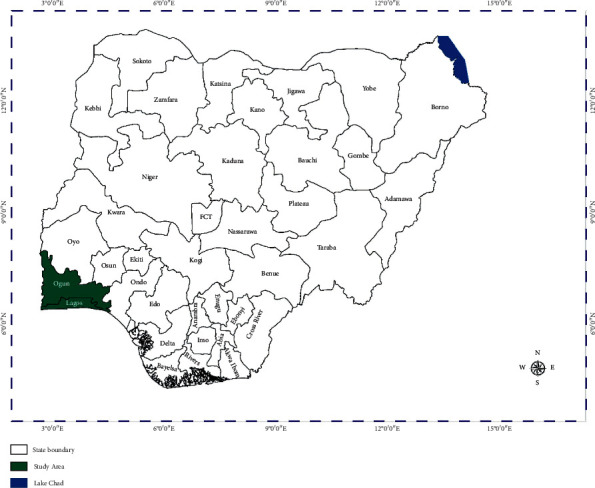
Map of Nigeria showing the study area.

The study was carried out in Lagos state (6^o^52ʹ44ʺ N, 3.37ʹ92ʺ E) and Ogun state (6° 90ʹ 75ʺ N 3° 58ʹ 13ʺ E), located in Southwest Nigeria (Figure 1). In Southwest Nigeria, three tiers of health care services are practiced, which are the tertiary, secondary, and primary healthcare systems [21]. These hospitals provide subsidised health care services to the masses. Massey Street Children Hospital is a children's referral hospital owned by the Lagos State government [22]. Orile-Agege General Hospital has an excellent hospital-based record of paediatric cases and immunisation services. State Hospital Ota is proxy to the study centre. Federal Medical Centre Abeokuta is a federal government-owned hospital with high patronage and also serves as a referral hospital in Ogun state [23, 24].

The study was a cross-sectional case-control investigation involving four local government areas (LGAs) in Lagos and Ogun state, which were Lagos Island and Agege LGAs from Lagos state, Ado/Odo Ota, and Abeokuta South LGAs from Ogun state (Figure 1), respectively. Participants included a total of 280 children comprising 143 children with acute diarrhoea (cases) and 137 children who were not suffering from diarrhoea (controls) over 12 months. The diarrhoeic children were those that have been diagnosed by the medical personnel at the hospital for this study, and the control groups were children within the same age bracket that were not having diarrhoea and were attending the same healthcare facilities.

## 3. Eligibility Criteria for the Study Participants

### 3.1. Criteria for Nondiarrhoeic Group (Control)

#### 3.1.1. Inclusion Criteria

  Under five children
  No acute diarrhoea
  Not on any antimicrobial treatment
  Informed written consent from the parents/guardians of children and assent from the subjects

#### 3.1.2. Exclusion Criteria

  Children whose parents did not consent

### 3.2. Criteria for Diarrhoeic Group (cases)

#### 3.2.1. Inclusion Criteria

  Under five children
  Acute diarrhoea (≥3 watery or loose stools with or without blood or mucus less than 14 days)
  Informed written consent from the parents/guardians of children and assent from the subjects

#### 3.2.2. Exclusion Criteria

  Children whose parents did not consent

### 3.3. Ethical Consideration

Participation was strictly voluntary. Parents/Caregivers received a thorough explanation of the aim, and objectives of the study were explained to the parents or caregivers. Each parent decided whether or not to participate or provide information about their children or wards. Participants were also enlightened about their freedom to withdraw in the course of the study without any consequences.

The ethical approval for this study was obtained from Covenant University Health Research Ethics Committee (CUHREC), Covenant University, Ota, Ogun State (CHREC/002/2018), Federal Medical Centre Research Ethics Committee (FMCREC), Idi-Aba, Abeokuta Ogun State (FMCA/243/HREC/03/2018/06), and Lagos State Health Service Commission (LSHSC/2222/VOL.1VB/232).

### 3.4. Questionnaire Survey

The authors developed a semistructured questionnaire. Mothers/caregivers responded through the assistance of hired research assistants. Communication was in English Language and local dialects where the respondents could not communicate effectively in the English Language. The questionnaires provided demographic, medical, nutritional, and environmental information about the children. Other information included the level of education and socioeconomic status of the mothers/caregivers.

### 3.5. Data Analysis

For quality control purposes, each completed questionnaire was proofed to avoid errors. The questionnaire data obtained were deposited in the SPSS Version 25 (IBM Corp., Armonk, NY, USA) program and Microsoft Excel 2013 for statistical analysis. Pearson's Chi square and *t*-test were used to determine the statistical significance in the relationships and or association between the dependent and independent variables. Comparisons were made between data derived from diarrhoeic and control subjects.

Logistic regression analysis was performed to assess the association of each predictor variable with the outcome variable. Measures of the relationship were expressed as crude odds ratios (CORs) for disease with 95% confidence intervals for variables.

Multivariable analysis was performed using all the significant variables obtained from the bivariate analysis. This provided the Adjusted Odds Ratios (AORs) at 95% confidence interval.

## 4. Results

Risk factors of diarrhoeal disease among children under five years of age in the study area.

The risk factors of the occurrence of diarrhoea among children below the age of five were categorised into three, namely: age and gender of the child, demographic and socioeconomic status of the mother, and medical history and environment of the child (Tables 1–6 ).

Table 1 shows the relationship between the age group and gender of the children and diarrhoea occurrence in the study area. These two variables were similar in both case and control groups, and there was no significant relationship between the age (COR = 0.359, CI = 0.101 – 1.274, *p*=0.113) and gender (COR = 1.245, CI = 0.774 – 2.003, *p*=0.366) of the children with diarrhoea occurrence in the study area.

Table 2 depicts the sociodemographic characteristics of caregivers or mothers for the case and control groups. All variables were significantly different between the case and control except for the age of the mothers. Results showed that there were significant relationships between diarrhoea occurrence and: mother's religion (OR = 0.185, *p*=0.0001), mother's educational status (OR = 11.459, *p*=0.0001), mother's employment status (OR = 2.082, *p*=0.025), and family income (OR = 7.613, *p*=0.0001).

Table 3 presents the medical history and environmental variables as risk factors for diarrhoea. There were significant relationships between where the child was born (OR = 0.221. *p*=0.009), mother's antenatal attendance (OR = 0.05, *p*=0.0001), exclusive breastfeeding (OR = 0.25, *p*=0.0001), reduced breastfeeding (OR = 4.3, *p*=0.002), and crèche attendance (OR = 2.04, *p*=0.011) and the occurrence of diarrhoea in this study. Children not exposed to drinking tap water (OR = 0.313, *p*=0.011), borehole (OR = 0.538, *p*=0.036), and sachets (OR = 0.214, *p*=0.0001) were less likely to have diarrhoea, whereas those that drank bottled water (OR = 2.548, *p*=0.001) were two times less likely to have diarrhoea (Table 3).

Tables 4 and 5 showed the values of the adjusted odds ratio (AOR) of the variables. Some of the variables shown to be statistically significant using crude odds ratios were also significant after being adjusted. Thus, the results revealed that the mother's level of education (AOR = 10.282, *p*=0.0001) and family income (AOR = 6.908, *p*=0.001) have a significant relationship with diarrhoea occurrence in children under five years of age (Table 6).

## 5. Discussion

This study identified three significant risk factors that could predispose children under age five to diarrhoea occurrence. These factors are: mother's educational status (OR = 11.46, *P*=0.0001), mother's employment status (OR = 2.08, *P*=0.025), and family income (OR = 7.61, *P*=0.0001). Other factors are the mother's antenatal attendance, duration of breastfeeding, child's crèche attendance, and source of drinking water.

The observation that children born by mothers with at most primary education is more likely to have diarrhoea than a child of a more educated mother supports other studies conducted in Lagos (Akinyemi, 2019), Osun, and Oyo States [25]. Literacy has been earmarked consistently as a major determinant of health in any population, especially with regard to female education. Educated women have a better understanding of personal hygiene, nutrition and are more knowledgeable about accessing the healthcare system. [26], concluded in one of their studies that maternal education is the most important risk factor to diarrhoea prevalence among children under five years of age in Nigeria.

Family income is a major risk factor of diarrhoea prevalence among children. This study revealed that a child from a family with reduced income is more likely to have diarrhoea than a child from a financially comfortable home. This study agrees with the findings of [19], in which low monthly household income had a significant association with childhood diarrhoea in Southwest Nigeria. Reference [25] concluded that low socioeconomic status impedes the health of children under age five in Southwest Nigeria. Global reports highlight the impact of poverty on the burden of diarrhoea disease prevalence among children [27, 28]. Reduced income subjects a family to poor living conditions such as lack of access to potable drinking water, improper sewage disposal, poor drainage system, and toilet facilities which have been identified as risk factors of childhood diarrhoea [29, 30]. The observation that children of unemployed mothers are twice likely to have diarrhoea than children of employed mothers (Table 2) is similar to findings of [31], which further corroborates the impact of poverty on childhood diarrhoea incidence. This implies that mothers generate more income for the family, thereby providing basic household sanitary materials that ensure hygienic living conditions for the children.

Bivariate logistics regression identified the mother's antenatal attendance, breastfeeding, and source of drinking water as protective factors of diarrhoea prevalence in children. Lack of access to potable water is a key player in the transmission of diarrhoeal diseases because unclean water harbours diarrhoeagenic pathogens. Studies from Southwest Nigeria have implicated faecal contamination of the water used for domestic purposes [32–35]. The level of microbial contamination of the water used for domestic purposes is higher in Lagos State than Ogun state because of the high population, thus leading to poor management of wastes, which could lead to pollution of water aquifers [32, 36]. This may be the reason for the high prevalence rate of diarrhoea in Lagos state (13.4%) than Ogun State (2%). Some of the drinking water (sachets) consumed in Lagos State are from unprotected sources [37, 38].

Exclusive breastfeeding confers immunity against diarrhoea in children below the age of five. This study revealed that children placed on exclusive breastfeeding were less likely to suffer from diarrhoeal diseases, while those fed with breastmilk for less than six months were more likely to have diarrhoea [39–41]. WHO and UNICEF recommend exclusive breastfeeding of infants for up to the first six months of life because of the nutritional and immunological value it provides to the child. Besides, breastfeeding helps to improve the cognitive and sensory development in infants. Besides, breastfeeding can protect a child against chronic and infectious diseases such as diarrhoea and pneumonia in children younger than five years of age. Breastfeeding has a huge impact on the health status of children. A significant correlation exists between breastfeeding and diarrhoea episodes [42, 43]. Suboptimal breastfeeding increases the risk of developing diarrhoea because breast milk can confer the proper functioning of the gut immune system in infants, both for a short- and long-term duration [44, 45]. Besides, breast milk contains antibodies, immunoglobulin A (IgA), which confer protection against pathogenic bacteria and harmonise the activity of white blood cells [46, 47].

## 6. Conclusion

This study strongly supports the fact that socioeconomic factors are significant determinants of childhood diarrhoea. Female education should be encouraged by the right government policy to achieve child favourable health outcomes in the future. Thus, the achievement of the sustainable development goal (SDG) 3 for a possible reduction of neonates and infants' deaths in Nigeria.

## Figures and Tables

**Table 1:** Association between child's age group, gender, and diarrhoea.

Characteristics		Diarrhoea status				COR (95% CI)	*P* value
		Cases		Controls			
		Number (n)	Percentage (%)	Number (n)	Percentage (%)		
Age group (Months)	0–11	64	44.76	102	74.45	0.359(0.101–1.274)	0.113
	12–23	51	35.66	20	14.60	1.457(0.384–5.525)	0.580
	24–35	12	8.39	6	4.38	1.143(0.237–5.501)	0.868
	36–47	9	6.29	5	3.65	1.029(0.199–5.326)	0.973
	48–59	7	4.90	4	2.92	1.00	
Gender	Male	64	44.76	54	39.42	1.245(0.774–2.003)	0.366
	Female	79	55.24	83	60.58	1.00	

*P* < 0.05 is significant; COR: crude odds ratios; CI: confidence interval.

**Table 2:** Association between demographic and socioeconomic variables of mothers/caregivers and diarrhoea.

Characteristics		Diarrhoea status				COR (95% CI)	*P* value
		Controls		Cases			
		Number (n)	Percentage (%)	Number (n)	Percentage (%)		
Mother's age (years)	<30	47	34.30	45	31.70	0.888 (0.539–1.464)	0.642
	>30	90	65.70	97	68.30		
Religion	Christianity	116	85.29	74	51.75	0.185 (0.104–0.329)	<0.0001
	Islam	20	14.71	69	48.25	1.00	
Educational status	Below tertiary	27	20.00	106	74.13	11.459 (6.521–20.139)	<0.0001
	Tertiary	108	80.00	37	25.87	1.00	
Employment	Unemployed	17	12.59	33	23.08	2.082 (1.098–3.950)	0.025
	Employed	118	87.41	110	76.92	1.00	
Family income	<100,000	70	61.40	109	92.37	7.613 (3.499–16.563)	<0.0001
	>100,000	44	38.60	9	7.63	1.00	
Residence	Duplex	5	3.68	1	0.75	0.043 (0.004–0.413)	0.006
	4-bedroom	7	5.15	2	1.49	0.061 (0.011–0.345)	0.002
	3-bedroom	17	12.50	8	5.97	0.101 (0.033–0.305)	<0.0001
	2-bedroom	65	47.79	19	14.18	0.063 (0.026–0.151)	<0.0001
	Self-contained	33	24.26	62	46.27	0.403 (0.175–0.928)	0.033
	Rooming house	9	6.62	42	31.34	1.00	
Toilet facility	Pit latrine	5	3.68	23	16.31	0.484 (0.084–2.783)	0.416
	Water closet	129	94.85	99	70.21	0.081 (0.018–0.355)	0.001
	None	2	1.47	19	13.48		1.00

(*P* < 0.05 is significant); COR: crude odds ratios; CI: confidence interval.

**Table 3:** The association between medical history and environmental variables and diarrhoea.

Characteristics		Controls		Cases		COR (95% CI)	*P* value
		Number (n)	Percentage (%)	Number (n)	Percentage (%)		
Where was your child born?	At home	2	1.46	11	7.69	1.375 (0.213–8.858)	0.738
	Hospital	131	95.62	116	81.12	0.221 (0.072–0.681)	0.009
	Traditional maternity centre	4	2.92	16	11.19	1.00	
How was your child born?	Caesarean section	34	24.82	26	18.44	0.685 (0.385–1.218)	0.198
	Natural birth	103	75.18	115	81.56	1.00	
Did you receive antenatal care?	Yes	135	98.54	107	76.98	0.050 (0.012–0.211)	<0.0001
	No	2	1.46	32	23.02	1.00	
Was baby breastfed?	Yes	137	100.00	142	99.30		
	No	0	0.00	1	0.70		
Was baby placed on exclusive?	Yes	105	77.21	65	46.10	0.253 (0.150–0.425)	<0.0001
	No	31	22.79	76	53.90	1.00	
If no, how long was the baby breastfed?	<6 Months	13	48.15	60	80.00	4.308 (1.677–11.065)	0.002
	>6 months	14	51.85	15	20.00	1.00	
Does baby attend a crèche?	Yes	27	19.85	45	33.58	2.041 (1.174–3.549)	0.011
	No	109	80.15	89	66.42	1.00	
Do you have a babysitter at home?	Yes	34	25.00	37	27.41	1.133 (0.659–1.947)	0.652
	No	102	75.00	98	72.59	1.00	
Tap water	No	130	94.89	122	85.31	0.313 (0.128–0.762)	0.011
	Yes	7	5.11	21	14.69	1.00	
Borehole	No	114	83.21	104	72.73	0.538 (0.301–0.961)	0.036
	Yes	23	16.79	39	27.27	1.00	
Well	No	137	100.00	143	100.00		
	Yes	0	0.00	0	0.00		
Sachet water	No	105	76.64	59	41.26	0.214 (0.128–0.359)	<0.0001
	Yes	32	23.36	84	58.74	1.00	
Bottled water	No	86	62.77	116	81.12	2.548 (1.480–4.387)	0.001
	Yes	51	37.23	27	18.88	1.00	
Boiled water	No	100	72.99	109	76.22	1.186 (0.692–2.033)	0.535
	Yes	37	27.01	34	23.78	1.00	
Rainwater	No	137	100.00	134	93.71		
	Yes	0	0.00	9	6.29		
River or stream	No	137	100.00	143	100.00		
	Yes	0	0.00	0	0.00		
Do you keep pets?	Yes	15	11.03	21	15.00	1.424 (0.700–2.893)	0.329
	No	121	88.97	119	85.00		

*P* < 0.05 is significant; COR: crude odds ratio; CI: confidence interval.

**Table 4:** Multivariate Analysis of mothers/caregivers' sociodemographic status.

Characteristics		Diarrhoea status		AOR (95% CI)	*P* value
		Controls (%)	Cases (%)		
Mother's age	<30	34.31	31.69	0.435 (0.179 – 1.056)	0.066
	>30	65.69	68.31	1.00	
Religion	Christianity	85.29	51.75	0.200 (0.070 – 0.569)	0.003
	Islam	14.71	48.25	1.00	
Educational status	Below tertiary	20.00	74.13	10.908 (40.62 – 29.291)	<0.0001
	Tertiary	80.00	25.87	1.00	
Employment	Unemployed	12.59	23.08	4.374 (1.453 – 13.165)	0.009
	Employed	87.41	76.92	1.00	
Income	<100,000	61.40	92.37	4.342 (1.364 – 13.821)	0.013
	>100,000	38.60	7.63	1.00	
Residence	Duplex	3.68	0.75	0.000	
	4 – bedroom	5.15	1.49	1.55 (0.10 – 2.398)	0.182
	3 – bedroom	12.50	5.97	1.540 (0.241 – 9.861)	0.648
	2 – bedroom	47.79	14.18	1.773 (0.320 – 9.832)	0.512
	Self-contained	24.26	46.27	2.157 (0.467 – 9.957)	0.325
	Rooming house	6.62	31.34	1.00	
Toilet facility	Pit latrine	3.68	16.31	NC	
	Water closet	94.85	70.21	0.153 (0.022 – 1.045)	0.055
	None	1.47	13.48	1.00	

(AOR: *p* < 0.005 is significant). AOR: adjusted odds ratios; CI: confidence interval.

**Table 5:** Multivariate Analysis of medical history and the environment as risk factors or diarrhoea in the study.

Characteristics		AOR (95% CI)	*P* value
Where was the baby born?	At home	5.381 (0.45963.063)	0.180
	Hospital	0.815 (0.185–3.603)	0.788
	Traditional maternity centre	1.00	
How was your child born?	Caesarean section	1.644 (0.730–3.699)	0.230
	Natural birth	1.00	
Did you receive antenatal care?	Yes	0.131 (0.026–0.659)	0.014
	No	1.00	
Was the baby placed on exclusive breastfeeding?	Yes	0.314 (0.154–0.641)	0.001
	No	1.00	
Does the baby attend crèche?	Yes	1.244 (0.599–2.583)	0.557
	No	1.00	
Is there a baby sitter or nanny at home?	Yes	0.746 (0.350–1.589)	0.447
	No	1.00	
Tap water	No	0.784 (0.238–2.584)	0.690
	Yes	1.00	
Borehole	No	0.252 (0.105–0.606)	0.002
	Yes	1.00	
Sachet water	No	0.130 (0.063–0.267)	<0.0001
	Yes	1.00	
Bottled water	No	2.658 (1.226–5.762)	0.013
	Yes	1.00	
Boiled water	No	1.128 (0.537–2.367)	0.751
	Yes	1.00	
Do you keep pets	Yes	0.971 (0.385–2.446)	0.950
	No	1.00	

(AOR: *p* < 0.005 is significant). AOR: adjusted odds ratios.

**Table 6:** Summary of Multivariate analysis of selected variables.

Characteristics	AOR (95% CI)		*P* value
Religion	Christianity	0.159 (0.055–0.462)	0.001
	Islam	1.00	
Education	Below tertiary	10.282 (4.369–24.198)	<0.0001
	Tertiary	1.00	
Income	<100,000	6.908 (2.266–21.055)	0.001
	>100,000	1.00	
Where was your baby born?	At home	5.280 (0.150–185.695)	0.360
	Hospital	0.893 (0.091–8.781)	0.923
	Traditional	1.00	
	Maternity centre		
Did you receive antenatal care during pregnancy?	Yes	0.418 (0.069–2.550)	0.344
	No	1.00	
Do you keep your baby in crèche?	Yes	2.418 (0.974–6.002)	0.057
	No	1.00	
Tap water	No	0.249 (0.046–1.343)	0.106
	Yes	1.00	
Sachet	No	0.213 (0.091–0.498)	<0.0001
	Yes	1.00	
Bottled water	No	1.400 (0.568–3.451)	0.465
	Yes	1.00	

(AOR: *p* < 0.005 is significant); AOR: adjusted odds ratios.

## Data Availability

The data supporting the results of this study are included in the manuscript.
